# Identification of Genes Promoting Growth of *Ustilago maydis* on Biomolecules Released from Cells Killed by Oxidation

**DOI:** 10.3390/jof8090957

**Published:** 2022-09-13

**Authors:** Jelena Malesevic, Milorad Kojic, Stefan Stanovcic, Natalija Azanjac, Mira Milisavljevic

**Affiliations:** Institute of Molecular Genetics and Genetic Engineering, University of Belgrade, Vojvode Stepe 444a, 11042 Belgrade, Serbia

**Keywords:** RUS, oxidative stress, genome protection, transcriptome

## Abstract

Much headway has been made in understanding the numerous strategies that enable microorganisms to counteract various types of environmental stress, but little is known about how microbial populations recover after a massive death caused by exposure to extreme conditions. Using the yeast-like fungus *Ustilago maydis* as a model, our recent post-stress regrowth under starvation (RUS) studies have demonstrated that this organism reconstitutes devastated populations with remarkable efficiency. Subsequently, we have identified four RUS-gene products. Two of these, Did4 and Tbp1, play parallel roles in protecting the genome. To identify additional molecular components, we took a molecular-genetic and a transcriptomic approach. By employing a simple and novel screening method, we identified five RUS-deficient mutants (*snf8*, *slm1*, *vrg4*, *snf5*, *hsf1*), three of which (*snf8*, *slm1*, and *hsf1*) displayed sensitivity to different genotoxic agents, indicating that the corresponding gene products have roles in genome protection. The global transcriptomic changes of cells grown in supernatants derived from peroxide-treated cell suspensions revealed sets of uniquely expressed genes. Importantly, among the genes induced by the substrates was *Chk1*, which encodes a protein kinase required for checkpoint-mediated cell cycle arrest in response to DNA damage. Mutants of *U. maydis* deleted of *Chk1* are severely incapacitated in RUS.

## 1. Introduction

Microorganisms possess an impressive variety of cellular mechanisms evolved to overcome various types of environmental stress, provided that the stressogenic factors do not occur in too great intensity, amount, or concentration. Yet, if the harmful conditions surpass the innate capacity to cope, the extreme stress exposure may cause accumulation of an overwhelming sum of cellular damage, leading to an excessive death and a dramatic decrease in population size. Consequently, the reconstitution of the population density in the wake of a massive death is a critical task. Given the prevalence and importance of microorganisms, the knowledge of the mechanisms and cellular factors underpinning population recovery is, thus, crucial for any comprehensive understanding of the strategies by which microorganisms persist even in strongly fluctuating environments.

It is principally within this conceptual and theoretical framework that our recent post-stress regrowth under starvation (RUS) studies have been developed [1,2,3]. Namely, we use the model basidiomycotan fungus *Ustilago maydis* to study the capacity of this single-celled haploid eukaryotic yeast-like microbe to reconstitute its populations after catastrophic stresses. To begin with, we employed the liquid holding (LH) assay system [4,5] to assess the ability of *U. maydis* to recover from heavy oxidative insults, discovering that the fungus has a remarkable capacity to recover from massive damage [1]. This initial investigation also established that the reconstitution of the devastated cell populations is promoted by growth and reproduction of the survivors, by feeding on the intracellular compounds leaked from the dead and dying cells. Importantly, the analysis of the growth effect of the substances released from the treated cells into the suspending medium revealed that the substrates have an opposing impact (nutritive and inhibitory) on the proliferation of freshly inoculated cells. On the one hand, the leakage products provide an accessible and rich supply of nutrients in quantities sufficient to support a robust multiplication of the inocula. However, increasing the dose of the stressors as well as prolonging the post-treatment incubation increases the inhibitory effect of the extracellular medium, which can be overcome by increasing the proportion of fresh to injured cells or by prolonging the time of LH incubation [1]. The observations thus indicate that *U. maydis* must possess and implement cellular operations involved not only in reabsorption of the released substrates but also in coping with their treatment-induced toxicity. Moreover, compared to *Saccharomyces cerevisiae*, *U. maydis* demonstrated evident superiority in effective processing and reuse of the leaked material, so that *S. cerevisiae* actually resembled some of the *U. maydis* RUS-mutants which were isolated as defective in performing RUS in peroxide-treated cell suspensions [2].

There are several other interesting aspects of RUS and the reader is referred elsewhere for an account of a broader theoretical background to the formulation of the biological meaning as well as to the articulation of the challenges and questions related to this phenomenon [3]. However, given that the exploration of RUS is still only in its pioneering phase, the elucidation of the molecular players and cellular operations involved in RUS is an issue that raises the most primary concerns. Indeed, it goes without saying that no microbial process can be adequately characterized, let alone understood, without actually furnishing sufficient information on the molecular underpinnings of the cellular operations supporting the phenomenon; at the very least, (micro)biological understanding requires knowing the factors involved. Therefore, the central focus of this study will be the identification of new cellular factors underlining RUS.

For precisely the same reasons, we have previously developed a screen for mutants defective in RUS, validated the screen by isolating a number of candidates, and characterized four of them (*adr1*, *did4*, *kel1*, *tbp1*) in considerable detail [1]. However, since the method employed in screening the mutants did involve multiple experimental steps, such as treating suspensions of cells of each individual candidate with different doses of peroxide, followed by determination of the surviving fractions for each of the reactions both on immediate plating as well as periodically over 3 days of LH incubation, the isolation of these mutants was a time-consuming and laborious process. Therefore, to be more effective we changed to using the supernatants derived from heavily treated (1% H_2_O_2_) cell suspensions as the substrate for a phenotypic screen for *U. maydis* mutants defective in RUS. This new, streamlined procedure was more efficient (less effort and time-consuming) than the previous one and resulted in the isolation of 33 mutants out of thousands readily screened. We have identified five of the mutants (*snf8*, *slm1*, *vrg4*, *snf5*, *hsf1*) and partially characterized their phenotypes. In addition to being necessary for recycling of the damaged intracellular compounds, Slm1, Snf8, and Hsf1 are also important for protecting the *U. maydis* genome against various genotoxins such as Ultravioletlight (UV), methyl methanesulfonate (MMS), and hydroxyurea (HU).

The investigation of the molecular factors involved in RUS may also be advanced and become more informative by the use of transcriptomics. So, we have performed global gene expression analysis through differential gene expression profiling of cells incubated in the supernatants derived from peroxide-treated cell suspensions vs. cells incubated in the rich growth medium. The transcriptome profiling revealed sets of uniquely expressed genes, and there was a positive correlation between the number of the exclusively expressed genes and the increasing toxicity of the substrates. Interestingly, among these genes was *Chk1*, encoding a protein kinase required for checkpoint-mediated cell cycle arrest, whose essential role in the maintenance of genome integrity has been extensively documented [6,7]. The ensuing gene function analysis of the *chk1* deletion mutant demonstrated very clearly that the gene is indispensable for the efficient growth of *U. maydis* cells on the substrates freed from the cells killed by oxidation.

Overall, by applying these two approaches, we identified a set of cellular factors, of which some are of unknown functions or with predicted roles in different cellular processes such as cell cycle regulation, stress response, endomembrane trafficking, etc. Again, overlapping roles with maintenance of genome integrity have been observed for some of the identified factors. In any case, the current study provides a new foundation for future efforts to understand the functional roles of these factors in RUS.

## 2. Materials and Methods

### 2.1. U. maydis Growth and Peroxide Treatment

Culture methods, transformations, gene transfer, and treatments of *U. maydis* have been described previously [8,9]. Nominal wild type strain UIMG10 (*nar1-6 a1b1*) was used for mutagenesis, treatments with clastogenes, and genetic analysis [10].

Cell number in liquid cultures was determined under microscope using a hemocytometer. *U. maydis* was grown in rich YEPS medium (1% yeast extract, 2% sucrose, and 2% peptone) at 30 °C with agitation at 200 rpm. Growth rates of wild type and mutants were determined as described in [1]. The results were analyzed by Student’s *t*-test using SPSS statistical software. *p*-values < 0.05 were considered significant.

Peroxide treatment was performed as described in [1]. Briefly, liquid cultures were washed three times with distilled water. An amount of 2 × 10^7^/mL of the washed cells was resuspended in 10 mM Fe^3+^-sodium EDTA and incubated at 30 °C for 10 min. Peroxide treatment was performed by adding H_2_O_2_ to initiate Fenton reaction, which was stopped after 10 min by adding distilled water and pelleted cells by centrifugation at 4 °C. After additional two washings, cells were resuspended in water at 2 × 10^7^/mL and plated immediately on a rich medium to measure survival or incubated at 30 °C with agitation and plated after 24–72 h to measure recovery. Spot assays were performed by making serial 10-fold dilutions from the initial cell suspension of 2 × 10^7^/mL and then spotting 10 µL aliquots of each dilution in sequence on solid medium. Plates were incubated for 3 days at 30 °C.

For the preparation of the supernatants, following the peroxide treatment, the cell suspensions were washed twice, resuspended in water at 2 × 10^7^/mL, and incubated in capped flasks mounted on an oscillating shaker at 30 °C for 4 h (or 16 h for the preparation of supernatants for the mutant screen). The cells were pelleted by centrifugation and the supernatants were passed through 0.45 µm-filters (Millipore, Burlington, MA, USA).

### 2.2. Mutant Screen and Gene Cloning

Exponentially growing cells of strain UIMG10 were spread on solid medium and irradiated with 254 nm UV light to a survival frequency of about 0.05%. Colonies arising from 3500 mutagenized survivors were tested for the ability to grow after incubation for 48 h in the cell-free supernatant derived from treated UIMG10 cells with 1% H_2_O_2_ in the presence of 10 mM Fe^3+^-sodium EDTA.

Five candidates with a loss of ability to grow were chosen for additional study (we will use the symbol “mir” to denote these mutants and it stands for mutants in RUS). The gene altered in mir3 was cloned by functional complementation of the MMS hypersensitivity after introducing a genomic DNA library prepared in a self-replicating vector with a hygromycin resistance marker as described previously [10]. mir2, mir7, mir27, and mir29 were cloned by three consecutive rounds of incubation in supernatant derived from treatment with 1% peroxide and incubated for a 48 h period in water. After each round of incubation in supernatant, the survivors were grown to saturation in a medium containing 100 µg/mL hygromycin. A total of 2 × 10^7^ cells was collected and incubated again through a subsequent round. After the third round, plasmids were extracted from survivors and the termini of cloned fragments were sequenced using primers from the vector. Usually, the genomic fragments are 5–15 kb long. Identity of the fragments was determined by matching with *U. maydis* genome sequence in the annotated JGI MycoCosm database (https://mycocosm.jgi.doe.gov; accessed on 4 February 2022). Candidate genes were subcloned to a single open reading frame (ORF) and retested for complementation. By sequencing of candidates ORFs, specific PCR amplicons were generated on genomic DNA of individual mutants and identities of mutated genes were determined: mir2—UMAG_11539 (Snf8), mir3—UMAG_03678 (Slm1), mir7—UMAG_01062 (Vrg4), mir27—UMAG_04381 (Snf5), mir29—UMAG_10368 (Hsf1).

### 2.3. RNA Extraction

For the analysis of the transcriptome, wild-type UIMG10 strain was grown over night and cells were washed two times. An amount of 2 × 10^7^ cells/mL was inoculated in 13 mL of YEPS, or cell-free supernatants derived from treatments with 0.4% or 0.7% peroxide, and inocula were incubated for 30 min at 30 °C with agitation. After that, the cells were collected and total RNA of each sample was extracted using the GeneJET RNA Purification kit (Thermo Fisher Scientific, Waltham, MA, USA) according to the manufacturer’s instructions with some modifications. Briefly, to increase the quantity of extracted RNA, the collected cells were resuspended in Yeast lyzing buffer (1 M sorbitol, 0.1 M EDTA, pH 7.4) containing 20 mM DTT and Lyzing enzyme from *Trichoderma harzianum* (Sigma, St. Louis, MO, USA). Suspensions were incubated for 30 min at 30 °C with agitation. Further steps were done according to the manufacturer’s instructions. DNase treatment was performed using DNA-free DNase Treatment and Removal Kit (Ambion, Austin, TX, USA).

### 2.4. Transcriptome Analysis

The total RNA purity, concentration, and quality were determined by an Agilent 2100 Bioanalyzer (Agilent Technologies, Santa Clara, CA, USA). mRNA enrichment, cDNA library preparation, 150 bp pair-end RNA-sequencing took place on an Illumina NovaSeq 6000, and data analyses were carried out by Novogene Bioinformatics Technology Co., Ltd., Beijing, China.

### 2.5. Data Analysis

Raw data were preprocessed to remove adapters, poly-N > 10% sequences and low-quality reads (Qscore of over 50% bases of the read ≤ 5) using the fastp tool. Clean data (clean reads) were obtained by removing reads containing adapter and poly-N sequences and reads with low quality from raw data. Clean data served for calculating of Q20, Q30, and GC content. All the downstream analyses were based on clean data with a high quality. Paired-end clean reads were mapped to the reference genome using HISAT2 software.

Featurecounts were used to count the reads mapped for each gene and expressed in FPKM (short for the expected number of Fragments Per Kilobase of transcript sequence per Millions base pairs sequenced), taking into account the effects of both gene length and reads count mapped to the gene [11].

Prior to differential gene expression analysis, for each sequenced library, the read counts were adjusted by Trimmed Mean of M- values (TMM) through one scaling normalized factor. Differential expression analysis of two conditions was performed using the EdgeR (without biological replicates) R package. The *p* values were adjusted using the Benjamini and Hochberg approach for controlling the false discovery rate. Genes with corrected *p* value < 0.005 and |log2^(Fold Change)^| > 1 found by edgeR were assigned as differentially expressed.

Enrichment analysis was performed using the clusterProfiler [12]. R package (with correction of gene length bias and threshold of corrected *p* value < 0.05) was used to test the statistical enrichment of differential expression genes in GO terms (Gene Ontology, http://www.geneontology.org/; accessed on 9 May 2022) and KEGG pathways (Kyoto Encyclopedia of Genes and Genomes; http://www.genome.jp/kegg/; accessed on 9 May 2022).

The data discussed in this publication have been deposited in NCBI’s Gene Expression Omnibus [13] and are accessible through GEO Series accession number GSE205897 (https://www.ncbi.nlm.nih.gov/geo/query/acc.cgi?acc=GSE205897; accessed on 17 June 2022).

## 3. Results

### 3.1. An Improved Method for Isolation of RUS-Deficient Mutants

We are interested in identifying the cellular factors involved in RUS. Since mutagenesis and isolation of mutants is one of the genetic approaches that offers effective ways to study (micro)biological processes and can in essence reveal the underlying molecular players involved, we opted for this methodology when we started to investigate the molecular basis of RUS [1]. Thus, to identify novel candidate factors required for RUS, we have devised a method that relied on mutagenesis and screening of the individual mutant candidates for their inability to recover after treatment with peroxide. As noted above, the procedure was labor-intensive since it included a number of steps starting with peroxide-treatments of the candidates, then followed by determination of the surviving rates in each reaction and determination of LH regrowth at 1-day intervals over 3 days. Moreover, we have (not uncommonly) encountered significant variability for the same mutants tested by successive treatments using the same dose of peroxide, perhaps due to the free-radical chemistry of the Fenton reaction, which required a lot of retesting. All in all, the efficiency of screening was low and of 1200 candidates, only 4 mutants were finally identified as RUS-defective. Put at its simplest, getting RUS-defective mutants in this way did indeed require a very time-consuming and labor-intensive effort.

Therefore, to increase the efficiency of isolation, we changed the screening method by taking advantage of the dual nature (nutritive and toxic) of the supernatants derived from heavily treated (1% H_2_O_2_) cell suspensions. For convenience, we will henceforth refer to these supernatants as “high dosage SN/s”. This change of the screening method was prompted by the simple observation that the mutants isolated by the previous method displayed sensitivity to high dosage SNs with such a difference from wild-type to promise rapid detection of mutants incapacitated in the recycling activity [1]. Indeed, this allowed us to greatly compress the whole process of the protocol. Hence, in the new procedure we mutagenized *U. maydis* wild-type cells, allowed them to form colonies, and tested these individually for loss of ability to grow in the high dosage SN. Thus, without the need for prior propagation in the liquid medium, individual colonies were directly inoculated in the SN and following 48 h incubation aliquots were plated on solid medium for determination of cell viability.

**Figure 1:**
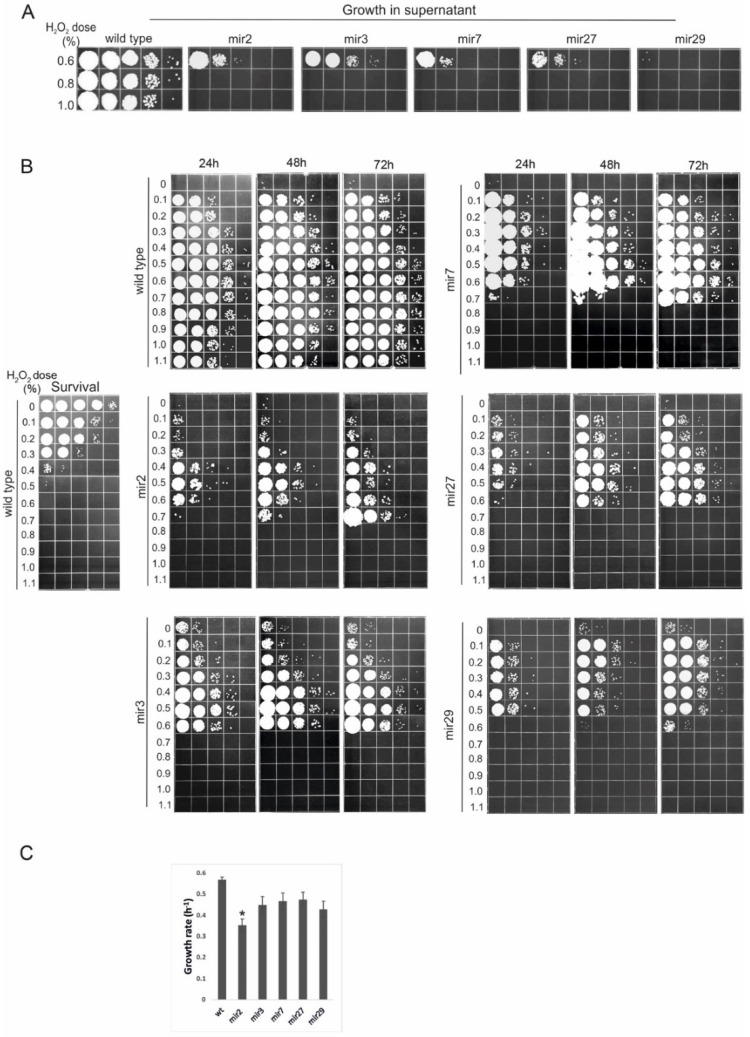
Mutants defective in RUS. (**A**) An amount of 8 × 10^3^ wild-type or mutant cells was inoculated in indicated supernatants and growth was examined after 48 h by spotting of ten-fold serial dilutions on a solid growth medium. (**B**) Growth of wild-type and mir mutant cells in cell-free supernatants derived from treatments of wild-type cells (4 × 10^7^) with increasing peroxide doses (left panel). (**C**) Growth rates of wild-type and mir mutants in complete medium. The error bars indicate standard deviations. Asterisks indicate significant differences (*p* < 0.05) between growth rates of each mutant and wild type. All experiments were performed at least three times.

Of 3500 mutants screened by this method, 33 showed the phenotype of interest, 5 of which had particularly compelling phenotypes and were chosen for more depth analysis. First, their incapacity to grow on toxic substrates was further confirmed by incubation in supernatants derived from high-dosage treatments with 0.6%, 0.8%, and 1.0% peroxide. An amount of 8 × 10^3^ wild-type or mutant cells was added to each supernatant and the growth was examined at 1-day intervals via the spot dilution assay. Figure 1A shows that the growth of all five mir mutants (“mir” for mutants in RUS) was totally inhibited in the high-dosage (0.8% and 1.0%) supernatants and that, except for mir29, all the other mutants were able to moderately proliferate in 0.6%-SN. Since the mutants have, in this preliminary analysis, shown a generally similar response, we decided to characterize their RUS-phenotype in more detail by assessing their growth response in a broader spectrum of supernatants (between 0 and 1.1%). Again, 8 × 10^3^ of wild-type or mutant cells was inoculated in each sample and the growth was examined at 1-day intervals (Figure 1B). Consistent with the observations described above, the complete lack of growth in high-dosage supernatants (above 0.7% peroxide) was common to all the mutants, which is unsurprising given the way the mutants were screened, i.e., by the absence of multiplication in the high-dosage (1%)-SN. However, in the lower-dosage SNs, each mutant showed a distinctive pattern of growth dynamics. Generally, as the toxicity of the substrates increased, each inoculum needed more time for proliferation. The most pronounced RUS-deficient phenotype was exhibited by mir2, with substantial growth lagging even in the low-dosage supernatants. The growth pattern of mir7 appeared comparable with that of wild-type within the range of 0.1–0.6%-SNs.

To exclude the possibility that the pattern of growth inhibition observed in mir mutants was the consequence of their generally reduced growth rates, we compared the growth rates of the parental wild-type strain and of the mutants incubating them in rich medium, YEPS. As shown in Figure 1C, only mir2 showed a statistically significant growth rate reduction, but the decrease was not drastic enough to explain even the pattern of growth inhibition exhibited by this mutant during 3 days of incubation in the low-dosage supernatants. Thus, we have compelling reasons to conclude that the dramatic growth inhibition observed in mir mutants was due to the disruption of the cellular operation/s other than those involved in the regular growth in rich medium. In all likelihood, the cellular factors corresponding to the genes altered in the mutants are all involved in the efficient processing and/or utilization of the toxic derivatives released from dead cells. By inference, this work also supports the conclusion that the method developed in the present study is indeed proven to be efficient and useful for isolating RUS-defective mutants. In fact, this is the first attempt to isolate a set of RUS-deficient mutants directly, based on their sensitivity to harmful components of the high-dosage SNs.

### 3.2. Response of Mir Mutants to Genotoxins

**Figure 2:**
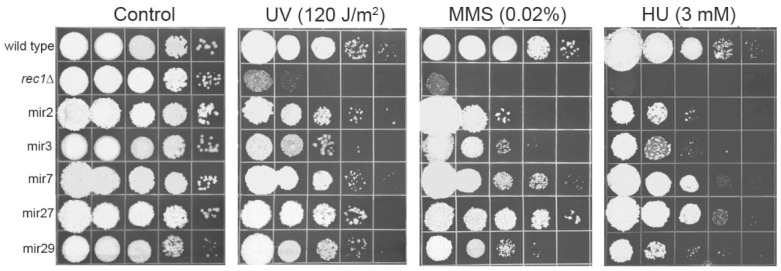
Response of mir mutants to genotoxic agents. Ten-fold serial dilutions of mutants were plated on solid medium then irradiated with indicated dose of UV or else plated on medium containing MMS or HU. The *rec1* mutant was used as a negative control, sensitive to all used agents. The testing was performed two times and representative results are shown.

Since all mutants exhibited enhanced sensitivity to cytotoxic compounds released from oxidatively damaged cells, it was logical to assume that at least some of them may also be sensitive to genotoxic agents. Indeed, such a finding would be in agreement with our previous observation that two of the RUS-defective mutants isolated through incubation in peroxide-treated cell suspensions had markedly pronounced DNA repair phenotypes [1]. Therefore, we tested these newly isolated mutants for their sensitivity to genotoxic agents, MMS, HU, and UV. Results are given in Figure 2. The wild-type strain UIMG10 was used as a positive, while *rec1* mutant (defective in a DNA damage checkpoint gene) served as a negative control. mir2, mir3, and mir29 showed mild sensitivity to MMS, and more pronounced sensitivity to HU. mir3 also showed mild sensitivity to UV. mir7 and mir27 exhibited marginal sensitivity to all genotoxins.

Thus, as inferred for previously isolated mutants [1], these findings reinforced the idea that at least some of the cellular factors important for efficient recycling of leaked, damaged intracellular biomolecules also function at the level of response to the DNA damage and replication stress. Sensitivity to MMS also can facilitate complementation cloning of genes by selection on this genotoxin.

### 3.3. Gene Identification

Identification of the mutated genes was performed by complementation cloning. The mutants were transformed with a wild-type genomic library prepared in a self-replicating plasmid. For the mir3 mutant exhibiting more pronounced sensitivity to MMS, selection of transformants for candidates resistant to MMS was done by replica plating onto agar plates containing 0.02% MMS. For the mutants with mild or no sensitivity to MMS, using this approach was not possible. Thus, for such cases we had to apply a different approach of selection, so we opted for three consecutive rounds of selective propagation in the high-dosage (1%)-SN. Transformants (~4000 colonies) were collected from a petri dish, expanded through one round of growth in YEPS, then allowed to proliferate for 48 h in the high dosage SN and then plated on solid medium. Transformants harboring a complementing DNA fragment were expected to grow in the wild-type manner, thus faster than the mutants. The process was repeated twice more, and after the final round of enrichment, randomly chosen individual colonies were tested for ability to multiply in the high-dosage (1%)-SN.

**Figure 3:**
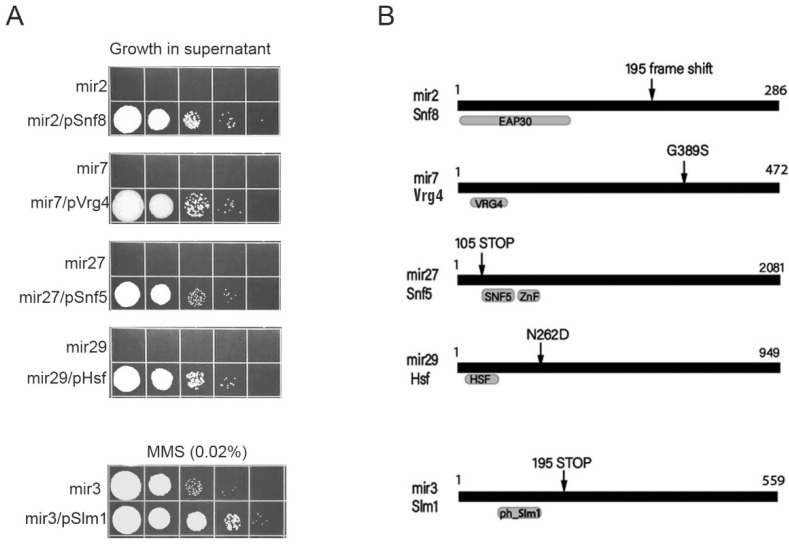
Identification of mir mutants and complementation. (**A**) Cells from the indicated strains transformed with a self-replicating plasmid containing the cloned gene were inoculated in supernatant derived from 1% treatment of wild-type cells and allowed to regrow for a 48 h period. All experiments were performed at least three times and representative results are shown. (**B**) Domain organization of identified proteins is shown schematically in black, with amino acid residue indicated. Protein classification and conserved domains are shown in grey. Protein identifiers in the JGI-annotated database are as follows: UMAG_11539 –Snf8; UMAG_03678 –Slm1; UMAG_01062 –Vrg4; UMAG_04381 –Snf5; UMAG_10368 –Hsf1. Arrows indicate sites of determined mutations.

Plasmid DNA was extracted from likely candidates and identities of the complementing inserts were determined by sequencing from either end. Candidates were subcloned when necessary to narrow down the complementing activity to a single open-reading frame. The complementation of the mutant phenotypes by recovered plasmids was confirmed for all five mutants (Figure 3A). For each mir mutant, one specific ORF was shown to complement the RUS-defective phenotype. To exclude the possibility of isolation of bypass suppressors, candidate genes in the mutants were sequenced in order to identify an inactivating mutation (Figure 3B).

**mir2** is defective in the gene coding for the 285 amino acid (aa) long hypothetical protein related to ESCRT-II complex subunit Vps22 (Snf8) protein [14,15]. The mutant allele results from a frame shift at codon 195 ensuing in the formation of a premature STOP codon. **mir3** carries a nonsense mutation causing a truncated version (195/559 aa) of the hypothetical protein homologous to yeast Slm1, a plasma-membrane-associated protein, a target of TORC2 and involved in a pathway regulating actin cytoskeleton organization in response to stress [16,17]. **mir7** is defective in the *Vrg4* gene encoding the 472 amino acid long putative GDP-mannose transmembrane transporter. The mutant allele results from a missense mutation leading to G389S amino acid change. The protein plays a role in transport of GDP-manose into the Golgi lumen where it serves for protein mannosylation [18]. The RUS phenotype of **mir27** is caused by a nonsense mutation in the gene encoding a 2081 aa-long hypothetical Snf5 subunit of the SWI-SNF chromatin remodeling complex [19,20]. Mutation creates a STOP codon at the N-terminus so that the predicted polypeptide, if expressed, would comprise the first 105 amino acid residues. **mir29** is defective in the gene encoding 949 aa-long Hsf1 (Heat shock transcription factor 1) [21]. The mutant allele results from missense mutation leading to N262D amino acid change.

### 3.4. Identification of RUS Genes through Transcriptome Analysis

Through an efficient and unbiased approach—mutant hunt—we have isolated a number of RUS mutants defective in a wide range of cellular functions. As an alternative approach that could accelerate the search for RUS cellular factors and give us a more comprehensive insight into an assortment of cellular operations important for post-stress recovery, we applied transcriptome analysis. One can expect that expression of genes important for RUS is changed (induced or decreased) in “unfavorable” conditions enabling cells’ better control over discrimination/selection of nutrient uptake (nutritive vs. toxic), their intracellular processing, and compartmentalization and protection of macromolecules from damaging agents. Additionally, the gene expression changes can depend on the level of substrate toxicity. So, we again took advantage of the dual nature of the supernatants and compared expression profiles of cells incubated in the supernatants obtained from treatments with two doses of peroxide, 0.4% and 0.7%, which differ in their toxicity level, versus cells grown in rich medium. Details on data statistics are given in Table 1, Figure S1, and https://www.ncbi.nlm.nih.gov/geo/query/acc.cgi?acc=GSE205897, accessed on 17 June 2022.

**Figure 4:**
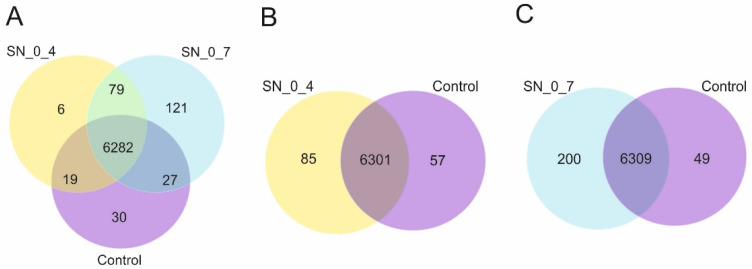
Venn diagrams of coexpression analysis (**A**–**C**). Numbers in colored circles presents number of genes uniquely or coexpressed in three samples. SN_0_4: cells incubated in supernatant derived from treatment with 0.4% peroxide; SN_0_7 cells incubated in supernatant derived from treatment with 0.7% peroxide; Control: cells incubated in diluted rich medium. Figures are provided by Novogene Co Ltd. and modified.

Venn diagrams of coexpression analysis are presented in Figure 4. Most of the genes (6282) are coexpressed in all three samples, thus, in cells grown under optimal, as well as under “risky” conditions. Evidently, there is a correlation between number of uniquely expressed genes and the increasing toxicity of the substrate. Cells grown in supernatant derived from the 0.4%-treatment (sample SN_0_4) uniquely expressed 85 genes, whereas in cells grown in supernatants prepared from the 0.7%-treatment (sample SN_0_7) that number is 200, as compared to the control cells (Figure 4B,C). Astonishingly, even 121 genes are exclusively expressed in cells of SN_0_7, of which 86 are hypothetical genes with unknown functions, opening up the possibility that at least some of them are dedicated exclusively to overcoming the toxicity imposed by the substrate. These hypothetical genes are mainly lacking conserved domains and exhibit limited homology to uncharacterized proteins from phylogenetically related fungi. Among the genes with conserved functions, uniquely expressed in SN_0_7 are those involved in oxido-reduction processes, transmembrane transport, metabolic processes (alcohol dehydrogenase, serine hydrolase, aspartate decarboxylase), and three genes containing a helix-loop-helix DNA-binding domain (UMAG_05080, UMAG_02092, UMAG_05486). SN_0_4 and control cells uniquely express 6 and 30 genes, respectively (Figure 4A). Uniquely expressed genes in SN_0_4 include domainless hypothetical proteins with recognizable homology to fungal hypothetical proteins, as well as a putative glucose transporter (UMAG_10608) and alpha-mannosidase (UMAG_04305). Interestingly, UMAG_10853, annotated as an uncharacterized protein of 102 amino acids, shows no detectable homology to any protein in other species. As with all the genes that lack recognizable homologs in other species, elucidation of the function of the UMAG_10853 will be challenging. However, the fact that it is implicated in RUS may serve as a good starting point.

**Figure 5:**
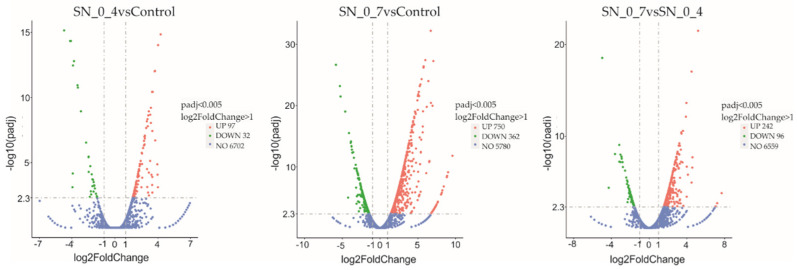
Volcano plot of distribution of differentially expressed genes. Horizontal axis: the fold change of genes in different samples. Vertical axis: statistically significant degree of changes in gene expression levels, the larger −log10(corrected *p*-value), the more significant the difference. The points represent genes, blue dots indicate no significant difference in genes, red dots indicate upregulated differential expression genes, green dots indicate downregulated differential expression genes. SN_0_4: cells incubated in supernatant derived from treatment with 0.4% peroxide; SN_0_7 cells incubated in supernatant derived from treatment with 0.7% peroxide; Control: cells incubated in diluted rich medium. Figures are provided by Novogene Bioinformatics Technology Co., Ltd.

The overall distribution of differentially expressed genes (DEG) is shown in Volcano plots (Figure 5). Again, cells of the SN_0_7 sample have the largest difference in gene expression profiles compared to the control in terms of not only the number of differentially expressed genes, but also in terms of the expression level of both up- and downregulated genes. Relative to the control sample, in SN_0_4 the total number of differentially expressed genes is 129, of which 97 are up- and 32 downregulated. Cells of SN_0_7 have a total of 1112 differentially expressed genes, of which 750 are up- and 362 downregulated. Compared to the SN_0_4 cells, the cells exposed to the more toxic substrate (SN_0_7) responded with a significant change in expression of 338 genes (242 being up- and 96 downregulated). In the SN_0_4, the level of induction of the up-regulated genes is up to 20-fold, in SN_0_7 the extent of induction for the up-regulated genes is more pronounced, so that for 42 genes it is above 100-fold. As in the case of induction, decreases in gene expression also occurred predominantly in SN_0_7 with a maximum decrease of nearly 57-fold, while the level of reduction in SN_0_4 was up to 26-fold. The greatest expression fold difference between SN_0_7 and SN_0_4 is approximately 30 times for both up- and downregulated genes.

**Figure 6:**
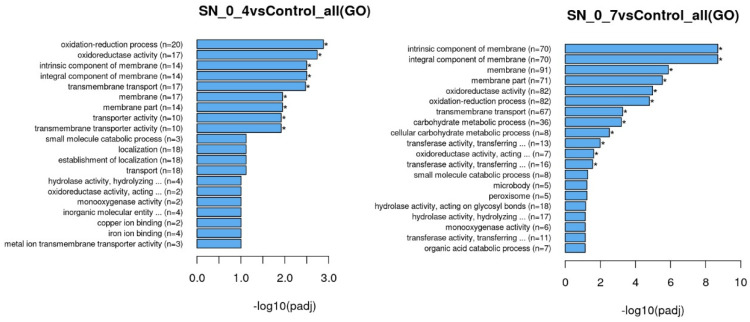
Gene Ontology (GO) Enrichment analysis of differentially expressed genes in SN_0_4 and SN_0_7 samples. SN_0_4: cells incubated in supernatant derived from treatment with 0.4% peroxide; SN_0_7 cells incubated in supernatant derived from treatment with 0.7% peroxide; Control: cells incubated in diluted rich medium. The length of bar charts indicates statistical significance of each GO term (* *p* < 0.05). Figures are provided by Novogene Bioinformatics Technology Co., Ltd.

Functional analysis of differentially expressed genes was performed through GO and KEGG enrichment to assign their biological functions (Figure 6 and Figure S2). Since imposed stress on cells is of oxidative nature and given that the cells can multiply in the substrates, meaning that they must uptake nutrients to be metabolically active, it is hardly surprising that most of the differentially expressed genes of both samples have roles in oxidation-reduction processes, transmembrane and intracellular transport, localization, and metabolic processes of various biocompounds (Figure 6).

Since the mutant hunt revealed that a number of genes implicated in intracellular trafficking (*Did4*, *Snf8*, *Vrg4*) and stress response (*Hsf1*) significantly contribute to the efficient RUS, we were interested to see whether the expression of the genes belonging to these functional groups was changed under our experimental conditions. The analysis has shown that four genes involved in endosome formation (ESCRT complexes) are up- (UMAG_05282 and UMAG_10799) or down- (UMAG_04129 and UMAG_10561) regulated in the SN_0_7 sample. Under the same conditions, six heat-shock proteins are up-regulated (UMAG_06430 -Hsp104, UMAG_03881 -Hsp16, UMAG_02057 -Hsp90, UMAG_03791 -Hsp70, UMAG_11952 -Hsp80, UMAG_05831 -Hsp60). UMAG_06430 and UMAG_03881 are also significantly induced in SN_0_4.

### 3.5. Chk1 Is Indispensable for RUS

Interestingly, although one can expect that the cytotoxic, and thus potentially genotoxic, nature of the supernatants can trigger changes in the expression of some genes involved in DNA repair and/or cell cycle progression, these genes were underrepresented among differentially expressed genes (only several genes coding for hypothetical proteins, of which some were up- (the putative 8-oxoguanine glycosylase involved in a base excision repair (UMAG_01304)) and others downregulated (putative ATM (Ataxia Telangiectasia Mutated; Serine/Threonine Kinase) homolog (UMAG_15011) and putative Mlh1-DNA mismatch repair protein (UMAG_00274) (https://www.ncbi.nlm.nih.gov/geo/query/acc.cgi?acc=GSE205897; accessed on 17 June 2022).

**Figure 7:**
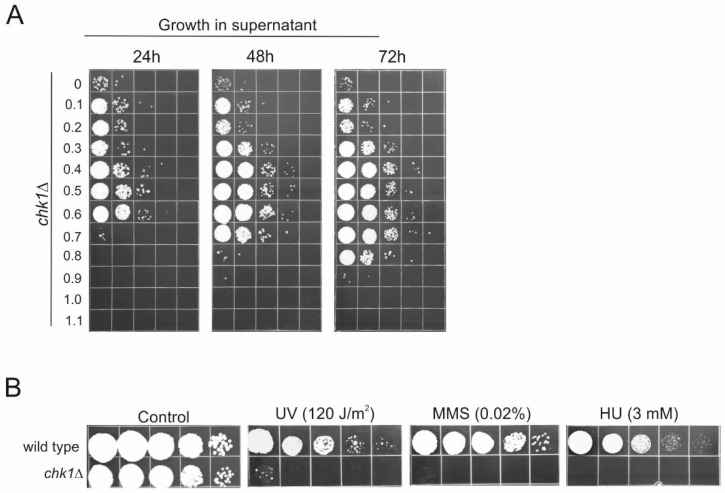
RUS phenotype of *chk1∆*. (**A**) Growth of wild-type and *chk1∆* mutant cells in cell-free supernatants derived from treatments of wild-type cells (4 × 10^7^) with increasing peroxide doses. Ten-fold serial dilutions were spotted on solid medium in 24 h-intervals. (**B**) Response of *chk1∆* to genotoxic agents. Ten-fold serial dilutions of mutant were plated on solid medium then irradiated with indicated dose of UV or else plated on medium containing MMS or HU. Experiments were performed three times and representative results are shown.

Among the upregulated genes was *Chk1* (UMAG_11087), known for its role in the protection of genome stability. Its expression is moderately elevated (2.8 times, log_2_^(Fold Change)^ = 1.5) in SN_0_7, compared to the control cells. *Chk1* encodes for a serine/threonine-protein kinase involved in the regulation of cell cycle progression and repair of DNA damages [6,7]. So, it was of considerable interest to determine whether Chk1 is indeed functioning in RUS. Therefore, we generated a null mutant and tested it for the ability to grow in the whole spectrum of supernatants (between 0 and 1.1%). The answer was compellingly affirmative. Namely, relative to wild-type, the *chk1∆* exhibited an unambiguous pattern of growth inhibition (Figure 7A). Evidently, the mutant was not only lagging behind wild-type across the entire spectrum of supernatants but was also completely inhibited in growth when inoculated in the highest-dosage supernatants. Evidently also, in the supernatants derived from cell suspensions treated with peroxide concentrations >0.7%, not just the absence of growth but even dying off of the inoculated *chk1∆* cells occurred. In the 0.7% SN, the mutant experienced a 1-day lag period (and initial loss of viability) before growth could pick up under these conditions. In the case of the 0.8% SN, the lag was extended over 2 days. In sum, the general conclusion was that deletion of *Chk1* conferred a clear growth disadvantage for its carries under the challenging (RUS) conditions. As expected, *chk1∆* is highly sensitive to genotoxic agents, UV, MMS, and the replication stressor, HU (Figure 7B). All in all, the finding increased the confidence in this approach taken to identify genes involved in RUS.

## 4. Discussion

This study was set in motion by the principal aspiration to improve biological understanding of RUS, which essentially requires further elucidation of the molecular basis of this phenomenon. Indeed, the identification of genes promoting the effective recycling of the damaged and liberated biomolecules is the key to any greater understanding of this major component of RUS. Therefore, to accomplish this objective we have undertaken a forward mutagenesis based on the new screen described above and carried out a comparative transcriptome analysis of *U. maydis* cells grown in YEPS and in the supernatants derived from peroxide-treated cell suspensions.

Employing the new screen, we isolated 33 mutants that display the RUS-deficient phenotype and so encode factors that may have roles in the processing and reusage of the toxic derivatives released from dying cells. We have identified five of the mutants (*snf8*, *slm1*, *vrg4*, *snf5*, *hsf1*) and partially characterized their phenotypes. In addition to being required for recycling of the damaged and released intracellular compounds, Slm1, Snf8, and Hsf1 are also involved in the protection of the *U. maydis* genome against various genotoxins such as UV, MMS, and HU. This is in line with our previous findings, which have found that two of the mutants isolated by the previous method (*did4* and *tbp1*) exhibited a particularly strong DNA-repair phenotype [1].

Prior to this investigation, we were operating with a rudimentary knowledge of the molecular players involved in RUS. Nevertheless, it was already apparent that the global cellular machinery required for RUS might actually be richly structured. Namely, the four cellular factors identified by our previous research (Adr1, Did4, Kel1, and Tbp1) had already been known to play roles in growth regulation, protein turnover, cytoskeleton structure, and transcription, indicating an assortment of the cellular functions underlying RUS. By now, these initial molecular insights are further exemplified by the variety of the molecular players identified by the new screen and, indeed, fully validated by the range of the cellular operations uncovered by the genome-wide transcriptome profiling (discussed below). Thus, RUS is evidently a very complex cellular operation that integrates a multiplicity of diverse molecular players and cellular processes. This conclusion is all the more in order given that our studies have identified several points of intersection between RUS and the maintenance of genome integrity. Of prime importance here is to elucidate the way in which these RUS factors contribute to the protection of the *U. maydis* genome.

The reciprocal angle of entry into this issue is from the perspective of genome protection. Namely, through the prism of transcriptomics and the subsequent gene-function analysis we have found that the checkpoint kinase 1 is indispensable for rendering *U. maydis* cells effective in RUS. The finding that Chk1 is so importantly linked to RUS does indeed provide a reason for interrogating this connection further. Since Chk1 plays a major role in the checkpoint-mediated cell cycle arrest in response to DNA damage [6,7], the finding calls for a detailed investigation of the DNA repair factors (primarily those known to be under direct mediation by the Chk1 kinase) for their potential role in RUS. Given that the leakage from the dying cells may provide not only beneficial but also harmful (genotoxic) molecules—for instance, oxidized nucleotides—and given that the survivors must then cope with this challenge of energy-rich but risky (mutagenic?) compounds, it would certainly not be surprising if some of the DNA-repair proteins are required for the efficient RUS. To recap, whichever angle we viewed it from, the results suggest that RUS and the genome protection are linked into a unified operation that is an effective vehicle for regrowth/repopulation after devastating stress.

Of note, through transcriptome analysis, we found no changes of expression of any DNA-repair genes in cells grown in the SNs, except a gene for the putative 8-oxoguanine glycosylase involved in a base excision repair (UMAG_01304). Moreover, a decrease in the level of gene expression was detected for putative ATM (Ataxia Telangiectasia Mutated; Serine/Threonine Kinase) homolog (UMAG_15011) and putative Mlh1-DNA mismatch repair protein (UMAG_00274). Probably, the DNA-repair gene transcripts/proteins are present in cells at a sufficient level and change in transcriptome profiles is not the best way for detecting them, as has also been shown for *S. cerevisiae* by [22].

Returning to the theme of diversity of the cellular factors involved in RUS, the conserved factor Snf5 is a component of SWI/SNF chromatin remodeling complex important for chromatin structure and transcription regulation from a variety of promotors [23,24,25]. With affected gene regulation, the yeast *snf5* null mutants display reduced growth on glucose and sucrose, are unable to grow on raffinose, galactose, or glycerol, and are hypersensitive to lithium and calcium ions [26,27,28]. Additionally, Snf5 senses nucleocytoplasmic pH oscillations and induces transcriptional reprograming under carbon starvation [29]. As a broad transcriptional regulator, it is not surprising that Snf5 is required for growth using damaged biomolecules. The same can be said of the hypothetical protein, homologous to Hsf1, a heat-shock transcription factor which is an activator of multiple genes in response to highly diverse stresses, including genes involved in protein folding, detoxification, energy generation, carbohydrate metabolism, and cell wall organization [30,31]. Importantly, the transcriptome analyses revealed seven heat shock proteins (Hsp 104, 16, 60, 70, 80, 90) upregulated in the higher-dosage SN, suggesting that the substrate-induced expression of these molecular chaperones and/or modifiers of proteostasis is at least partly responsible for enabling the cells for the optimal RUS. In other words, the upregulated expression of these factors that can elevate the cellular capacity for protein folding, preclude proteins from aggregating, facilitate aggregate dissolution, or suppress protein toxicity [32,33,34] not only implies that the cells growing under RUS conditions do experience stress, but also suggests that they likely use the aggregate dissolution coupled with degradation to clear the cytoplasm of potentially toxic species. Further studies are required to address these possibilities. Interestingly, we found that transcription of the *Hsf1* gene, whose product in *S. cerevisiae* activates transcription of a set of Hsp genes [35,36], was not affected during incubation in the suspensions. In both mutant hunts, through cell suspension and supernatants, we isolated mutants in endosomal trafficking, component of ESCRT-III (Did4), and component of ESCRT-II complex (Snf8/Vps22), respectively. Additionally, two genes coding for components of ESCRT-III (related to Snf7) were upregulated in cells grown in SN_0_7. This indicates that proper processing and recycling of retaken damaged compounds (for instance, recaptured oxidized peptides) is crucial for the multiplication on these harmful substrates. Similarly, endosome/multivesicular bodies formation and activities are necessary for the repair of membranes, which can also be critical for the stress-survivors to restore their membranes’ integrity after massive oxidative stress.

It is indeed worthy of note that actually none of the genes identified via previous or through the current screen for RUS-defective mutants were detected by our transcriptomic analysis as up- or downregulated. The simplest explanation for this would be that the genes are constitutively expressed. Another possible explanation is that the finding might be related to the time point chosen for the mRNA extraction. Namely, it is possible that some of the SN-responsive genes are induced only at later phases of growth in the SNs. Transcriptional analysis of *U. maydis* genes at different time points during growth in the SNs will be useful in further understanding the global response in this RUS-competent organism. This is not a unique example of the lack of correlation between specific mutant fitness decrease and the increased gene expression. Comparison of expression and fitness profiling of yeast grown in altered environmental conditions (1 M NaCl, 1.5 M sorbitol, pH 8.0 and galactose) showed that less than 7% of the genes whose inactivation significantly affected growth in galactose-containing medium were also upregulated under the same conditions. Even more, in the case of pH 8.0, 1 M NaCl, and 1.5 M sorbitol, only 3.0%, 0.88%, and 0.34%, respectively, of the genes that showed a significant increase in mRNA expression also exhibited a significant decrease in fitness [37,38]. This can be the consequence of gene redundancy, and although many genes are upregulated, only a small fraction is essential for the process.

All this may suggest that the exclusive use of transcriptomic profiling to detect the genes underlying RUS may overlook the constitutively expressed regulatory genes that play crucial roles in establishing tolerance limits. Thus, like any other experimental approach, transcriptomics is associated with some limitations. On the other hand, we have a random mutagenesis and isolation of mutants methodology as one of the powerful tools for gene function discovery that provides several clear advantages. First of all, the functions of genes that are constitutively expressed can be evaluated. Another advantage is that mutational alternations tend to produce reliable phenotypic changes suitable for quantitative analysis. Additionally, a cause–effect relation between the mutation and the phenotype can even suggest mechanisms of the wild-type gene action. However, there are several disadvantages, too. First, the approach is less systematic and does fundamentally depend on chance. Another disadvantage is that essential genes may frequently be excluded. Additionally, given that the genes involved in RUS are scored by identifying loss of function mutations, any genes that have redundant functions are unlikely to be identified. Therefore, we would argue that the combination of these approaches would improve the outcomes. The results that have been accumulated in this study make it easier to agree.

Time-series gene expression profiling, expression of non-translated RNAs, changes in the proteome and metabolome profiles, post-translational modifications, and changes in protein localization could also be aspects of post-stress strategies of cell survival and can be the subject of some future investigations.

## 5. Conclusions

The research described in this manuscript draws on work previously reported from our laboratory on the post-stress restitution of viability in the decimated populations and on the isolation of *U. maydis* mutants defective in RUS. Yet, prior to this study, we had only a basic knowledge about the molecular factors supporting this process. Therefore, the principal concern of this work was to better understand the molecular bases underlying this adaptation. Here, we report the successful identification and partial characterization of a new set of RUS-defective mutants. This was achieved through a new screening approach that employed high-dosage SN. We have also extended our studies further by analyzing the global gene expression, which revealed sets of uniquely expressed genes. The involvement of the heat-shock proteins and of several components of the endocytic/endosomal trafficking-associated processes, as well as of membrane components plus the proteins involved in oxido-reduction processes, emerged as particularly important. Thus, the progress made in this study is significant and provides a broadened scope for future RUS research. However, particularly attractive and plausible is the intersection between RUS and the protection of genome integrity. The hope is that the impact of the future studies may be the emergence of a new perspective, of new insights and understanding.

## Figures and Tables

**Table 1:** Summary of RNA-seq data.

Sample Name	Raw Reads	Clean Reads	Raw Bases	Clean Bases	Error Rate (%)	Q20 (%)	Q30 (%)	GC Content (%)	Total Mapping Rate (%)	Uniquely Mapping Rate (%)
Control	29,712,747	29,540,513	8.9G	8.9G	0.02	98.1	94.4	56.29	96.51	96.08
SN_0_4	28,472,163	28,298,524	8.5G	8.5G	0.02	98.1	94.2	56.38	96.66	96.17
SN_0_7	31,968,665	31,618,485	9.6G	9.5G	0.02	98.5	95.6	56.16	96.27	95.63

## Supplementary Materials

The following supporting information can be downloaded at: https://www.mdpi.com/article/10.3390/jof8090957/s1, Figure S1. Gene expression level analysis provided by Novogene Bioinformatics Technology Co., Ltd.; Figure S2. KEGG enrichment of differentially expressed genes provided by Novogene Bioinformatics Technology Co., Ltd.
