# Supplementary material for: Identification of Genes Promoting Growth of Ustilago maydis on Biomolecules Released from Cells Killed by Oxidation
Source: J Fungi (Basel). 2022 Sep 13;8(9):957. doi: 10.3390/jof8090957 (PMC9503528; doi:10.3390/jof8090957)

Figure S1. Gene expression level analysis

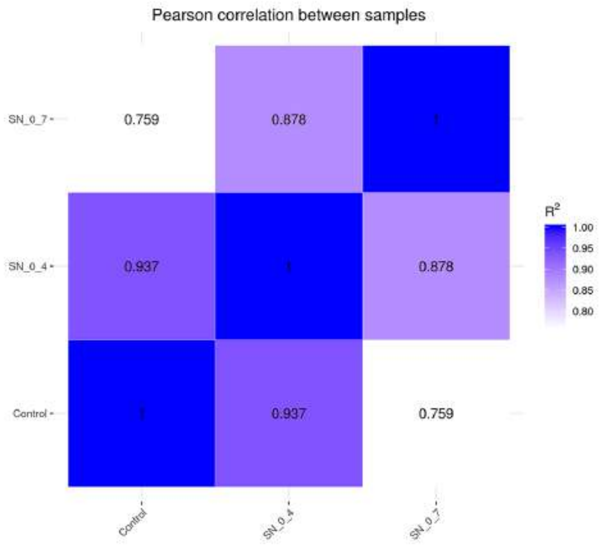

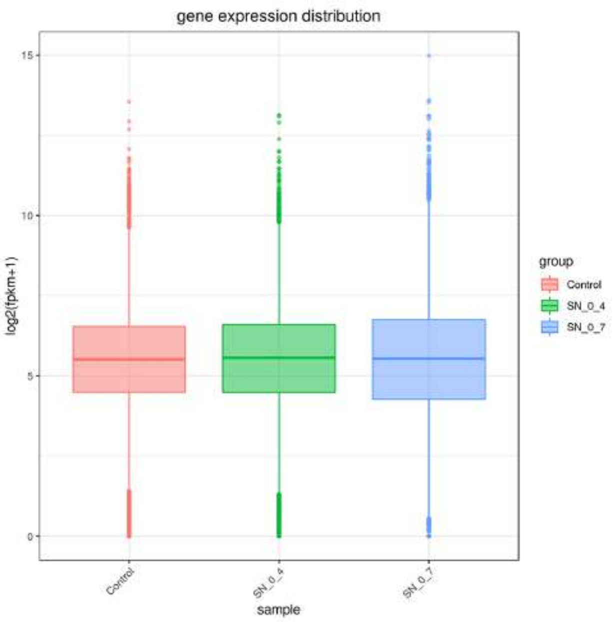

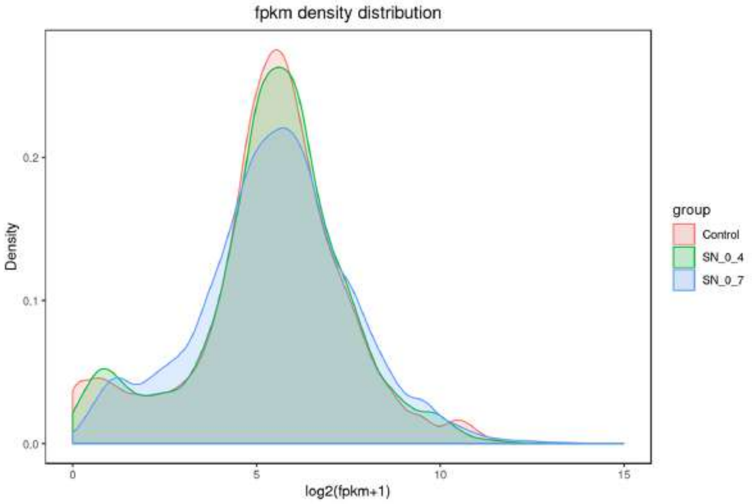

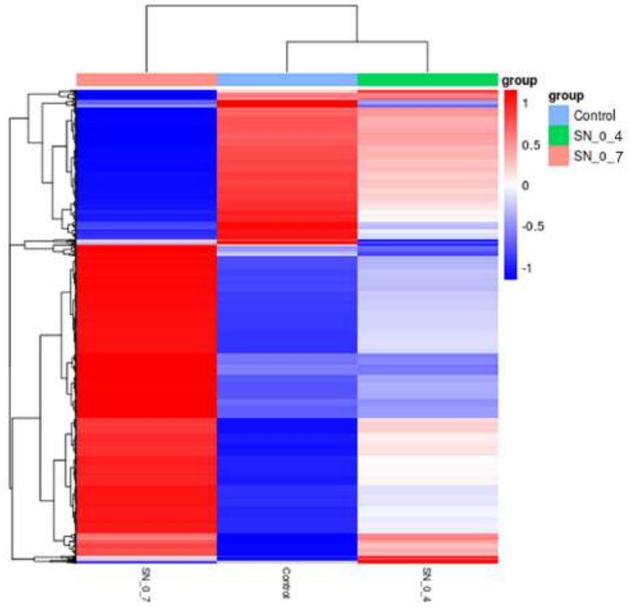

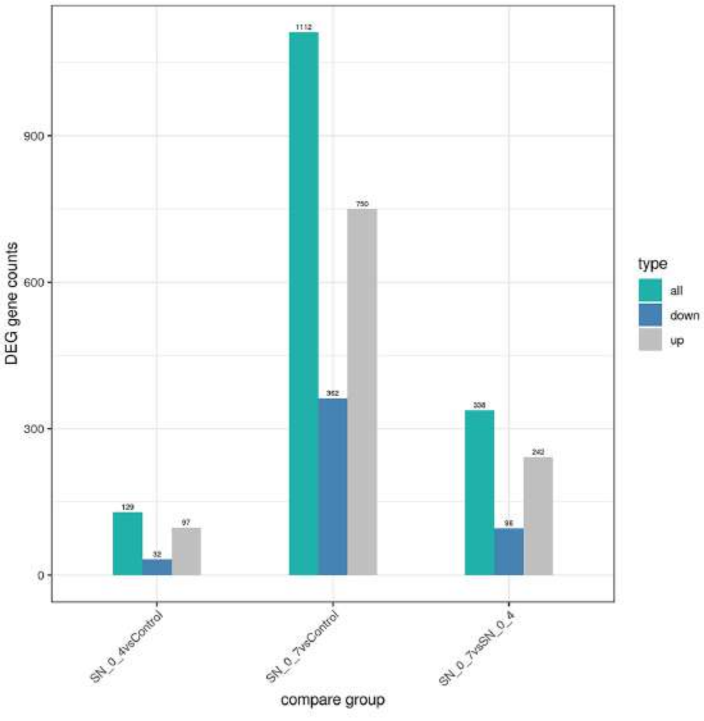

Figure S2. KEGG enrichment of differentailly expressed genes

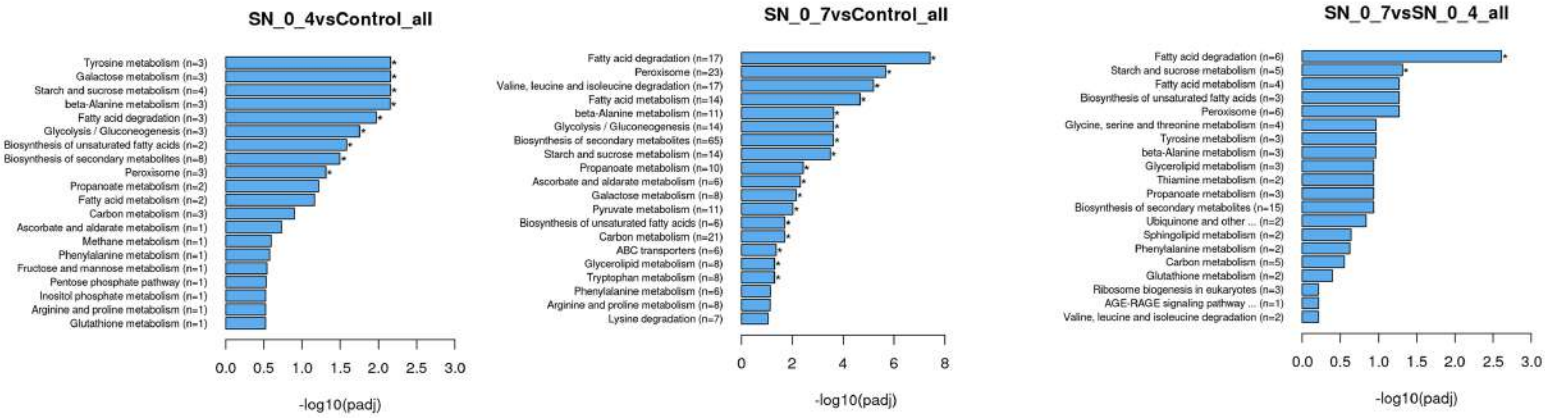

Supplement: Supplementary file 1 [file jof-08-00957-s001.zip › jof-1852215-supplementary.pdf]
